# Editorial: Dynamic photosynthesis under non-steady conditions

**DOI:** 10.3389/fpls.2024.1489818

**Published:** 2024-10-18

**Authors:** Silvere Vialet-Chabrand, Shizue Matsubara, Tracy Lawson

**Affiliations:** ^1^ Wageningen Plant Research, Wageningen University and Research, Wageningen, Netherlands; ^2^ IBG-2: Plant Sciences, Forschungszentrum Jülich, Jülich, Germany; ^3^ School of Life Sciences, University of Essex, Colchester, United Kingdom

**Keywords:** photosynthesis, fluctuating light intensity, acclimation (photosynthesis), gas exchange modelling, abiotic stresses

Photosynthesis occurs under fluctuating environmental conditions in the field and greenhouse growth environments and rarely reaches a steady state. Compared to the conditions used for most laboratory measurements, leaves within a crop canopy experience rapid fluctuations in microclimatic conditions due to the canopy structure (e.g. self-shading), clouds passing, and wind gusts, resulting in temporal and spatial heterogeneity in physiological responses. Plants growing under non-steady-state environmental conditions acclimate to the environment impacting photosynthetic responses, plant growth, development, and yield. Over the last decade, the scientific community has accumulated evidence from several crop species that acclimation responses to light fluctuations, relative humidity, and temperature greatly impact plant processes and our understanding of these is still limited. This Research Topic focuses on short (within minutes) and long-term (within days) photosynthetic responses to dynamic environmental conditions. To this aim, this Research Topic covers manuscripts that explored how short-term photosynthetic responses differ in C_3_ vs C_4_ plants (Arce Cubas et al.) and among genotypes (Zhang et al.; Burgess et al.). Additionally, the long-term influence of salinity on leaf gas exchange (Mousavi et al.), as well as the impact of combined environmental fluctuations in light intensity and temperature on crop growth are investigated (Zepeda et al.). In this exploration, dynamic photosynthesis modelling is a useful tool to interpret acclimation mechanisms that are often difficult to disentangle from environmental fluctuations (Salvatori et al.).

Photosynthetic induction is the process by which leaves begin to increase the assimilation of CO_2_ once they transition from low to high light intensity (Acevedo-Siaca et al, 2021) and is characterized by a lag in efficiency due to the regeneration of Ribulose 1, 5-bisphosphate (RuBP), the buildup of carbon metabolite intermediates, activation of Ribulose 1, 5-bisphosphate carboxylase/oxygenase (Rubisco), and stomatal opening as photosynthesis moves toward a steady-state (Pearcy et al., 1994; Mott et al., 2000). In this Research Topic, Zhang et al. quantified the genotypic variation of photosynthetic induction in 19 genotypes among six horticultural crops. They observed variations in photosynthetic induction kinetics that were greater between crops than between cultivars of the same crop. The time taken to reach 20–90% of full *A* induction varied by 40–60% across genotypes, and this was driven by variation in stomatal opening rather than Rubisco activation kinetics. There is ongoing debate regarding the primary mechanism(s) limiting photosynthetic induction, with studies on various species reporting diverse limitations. Each mechanism involved in photosynthetic induction likely imposes a limitation at different times of the day depending on the environmental conditions.

In the literature, the C_4_ carbon concentrating mechanism (CCM) is often presented as a major improvement in efficiency over C_3_ pathways. Arce Cuba et al. suggested that although this is true under steady-state conditions, under fluctuating light intensity the CCM may negatively affect photosynthetic induction due to a loss of coordination between the C_3_ and C_4_ cycles in C_4_ species. Their results showed that C_4_ species have slower activation of CO_2_ assimilation during photosynthetic induction than C_3_ species, but the apparent mechanism behind these differences varied between genera. The large variation in efficiency observed suggested that the CCM could be an unexploited breeding target for better performance under dynamic lighting intensity.

Under continuously dynamic light intensity, the induction of photosynthesis limits carbon gain and the fluctuation patterns over multiple days determine the degree of photosynthesis limitation (Matthews et al., 2018). Burgess et al. showed that acclimation of light harvesting, photosynthetic capacity and dark respiration are controlled independently under fluctuating light environments. They studied the acclimation potential of contrasting *Arabidopsis thaliana* genotypes, and although light history influences the capacity to acclimate to a change in irradiance, the length, or speed, of response to light history is also genotype-specific. Our knowledge about acclimation to fluctuating light intensity is still growing and the empirical model proposed in this study highlights the need to work on a mechanistic model that will further our understanding of the processes involved. This was further emphasised in the study by Salvatori et al. who demonstrated similar steady-state photosynthesis but different biomass in a chlorophyll-deficient soybean mutant compared to wild-type, grown under different light patterns and low light intensity. Using a dynamic photosynthesis model, they hypothesised and found evidence that the mutant was less efficient under fluctuating light intensity due to photosynthesis induction mechanisms which over time reduced biomass. Overall, such studies highlight the role of modelling in building our understanding of the complex dynamic response of photosynthesis.

In their study, Zepeda et al. explored the impact of light intensity and temperature fluctuations on the synchronization between carbon supply by photosynthesis and carbon demand by plant organs. Combining fluctuations of two environmental variables is an important challenge to further our understanding of crop responses to a fluctuating climate, which is not often reported in the literature. Their results supported that storage and remobilization of non-structural carbohydrates (NSC) are important processes that allow plants to buffer environmental fluctuations. They conclude that growing plants under fluctuating conditions does not necessarily have detrimental effects on plant growth and may improve biomass production in some plants. It is important to note that in their experiments, fluctuations were defined over days, which shows that fluctuations at second, hour and day levels all together impact carbon gain.

Fluctuations in water availability are also important to consider in the field with intermittent rain and variable soil water capacity. In such conditions, crops may experience multiple short drought episodes that impact their plant morphology and leaf anatomy. According to Mousavi et al., growth under limited water availability due to salt stress led to specific acclimation responses (e.g. lower stomatal density and size) reducing water loss, which was genotype-dependent. These acclimation mechanisms are known to impact short-term responses in photosynthesis in interaction with other climate variables, which can have consequences on growth and yield.

This Research Topic only covers a small fraction of the possible combinations of environmental fluctuations occurring in the field. Furthermore, plant responses to environmental fluctuations can be altered under stresses, which leads to different acclimation responses that have yet to be studied to understand the complex interaction mechanisms involved. Our knowledge of dynamic plant acclimation often requires time-consuming experiments as acclimation is a slow process over multiple days. High throughput phenotyping studies focused on acclimation could help reduce our knowledge gap and could lead to further improvements in photosynthesis and yield in the future.
